# Caregiver mind-mindedness training as an early intervention for social anxiety in children: A protocol for a randomised controlled trial

**DOI:** 10.1371/journal.pone.0315150

**Published:** 2025-09-22

**Authors:** Hiva Javadian, Mary E. Stewart, Minu Mathews, Andrew James Williams, Daniel Hale

**Affiliations:** 1 Psychology Department, Heriot-Watt University, Edinburgh, United Kingdom; 2 Department of Psychology, Heriot-Watt University, Dubai, United Arab Emirates; 3 Scottish Collaboration for Public Health Research and Policy, University of Edinburgh, Edinburgh, United Kingdom; Federal University of Ceara, BRAZIL

## Abstract

**Background:**

This paper describes a randomised controlled trial (RCT) protocol aimed at investigating the efficacy of caregivers’ Mind-Mindedness training as an early intervention for preschoolers with social anxiety. Mind-mindedness, a caregiver’s ability to recognise and respond to a child as an individual with their own thoughts, feelings, and intentions, is associated with secure attachment and socioemotional skills. While previous studies indicate brief Mind-Mindedness training may increase caregiver mentalisation in high-risk groups, there is a lack of rigorous evidence assessing its impact specifically on child social anxiety symptoms. Building on the well-established link between caregivers’ Mind-Mindedness and positive socioemotional outcomes in children, this study aims to bridge the existing research gap by directly testing the impact of Mind-Mindedness training on social anxiety.

**Methods:**

This randomised controlled trial aims to recruit 100 caregivers of preschool-aged 4–7-year-old children with social anxiety from the UK and Iran. The caregivers will be randomly assigned to either a mind-mindedness training group (n = 50) or a peer support (control) group(n = 50). The mind-mindedness training will involve three online sessions, each of one hour duration, focused on teaching strategies for using mind-minded comments with their children, across three consecutive weeks. The peer support (control) group will have access to a private online peer-support platform for sharing experiences. Measures of mind-mindedness, child social anxiety, attachment, and theory of mind will be assessed at baseline, post-intervention, and 3-month follow-up using established assessment tools.

**Discussion:**

The study aims to evaluate the effectiveness of a mind-mindedness parental intervention for social anxiety in children and to uncover the potential mediating roles of attachment and theory of mind in the relationship between mind-mindedness and child anxiety. The cross-cultural design, involving participants from the UK and Iran, will offer valuable information on the cultural aspect of the intervention. The training group is hypothesised to lead to increased mind-mindedness and reduced child social anxiety versus a peer support (control) group. This research can establish evidence for mind-mindedness training as an early intervention approach for childhood social anxiety, with implications for global mental health strategies targeting early caregiver-child relationships.

**Trial registration:**

Prospectively registered on ClinicalTrials.gov (ID: NCT06657014; registered on 23rd October 2024) and on the Iranian Registry of Clinical Trials (IRCT) Version 1 (ID: 80088; approved on 2nd November 2024). These registrations promote transparency and ensure compliance with international standards for reporting clinical trials.

## 1. Introduction

### 1.1. Background and rationale

Social anxiety is a common mental health concern in early childhood and can impact a significant number of children, particularly preschoolers, and has long-term mental health implications if left unaddressed [1–3]. Anxiety symptoms in young people have been exacerbated by the COVID-19 pandemic, highlighting the need for effective early interventions [4]. Social anxiety is characterised by feelings of distress and avoidance of social settings due to a fear of negative evaluation [5] which can impair the development of social relationships and pose challenges in adapting to school life [6,7]. Anxiety levels often begin to present between the ages of 2–4, likely due to significant life changes such as starting preschool [8,9].

It is also essential to understand that certain expressions of anxiety, such as separation anxiety, are considered typical during early childhood and often indicate normal developmental transitions, and are developmentally normative during this period. These symptoms frequently arise in response to significant milestones, including starting preschool, navigating new peer relationships, and experiencing temporary separations from caregivers [10,11]. It is crucial to distinguish between developmentally appropriate anxiety and clinically significant anxiety symptoms for early identification and intervention.

The preschool period is a critical stage in a child’s life, as it involves significant changes for the child, with changes to the teacher, peers, and environment. These changes can be challenging to navigate [12–14]. Parenting style and attachment are strongly linked to childhood anxiety [11,15]. Early intervention for preschoolers with social anxiety, through targeted approaches that focus on parenting and attachment, addressing caregiver behaviours as a key mechanism of influence, can help prevent symptoms from escalating into more severe disorders as the child develops [16,17]. The preschool years are, therefore, of critical importance for anxiety prevention efforts. While the impact of parenting style and attachment on a child’s anxiety is well-documented, interventions often focus exclusively on the child, neglecting the crucial role of caregiver behaviour [15].

One particular aspect of caregiver behaviour is Mind-Mindedness (MM). MM involves a caregiver’s ability to recognise and respond to a child as an individual with their own thoughts, feelings, and intentions [18,19]. Higher levels of MM are associated with secure attachment and positive socioemotional outcomes [18,20–23]. However, the direct impact of caregiver MM on child social anxiety remains underexplored. While the concept of MM is well-established in developmental psychology, its direct impact on child social anxiety has not been extensively explored in empirical research. Nevertheless, an increasing body of evidence indicates that MM has a causal influence on critical developmental mechanisms, particularly attachment [18,19] and Theory of Mind (ToM) [20,21,24,25] that are subsequently linked to anxiety outcomes.

MM is not just a descriptive ability; it represents a dynamic, relational process in which caregivers actively interpret and respond to their child’s internal states. Parents who exhibit higher levels of mind-mindedness by making appropriate and attuned comments about their child’s thoughts, emotions, and intentions have been shown to promote better emotional regulation, social competence, and executive functioning in their children (Meins, 1999; Bernier et al., 2010). These positive outcomes are likely shaped by more secure attachment relationships and enhanced ToM development, both of which act as protective factors for social anxiety [19,20,25].

Although MM has yet to be directly tested as an intervention for social anxiety, its known effects on attachment and ToM make it a promising candidate for early intervention. This study seeks to rigorously examine the efficacy of MM training for caregivers in reducing symptoms of social anxiety in children by reinforcing these foundational developmental processes.

Meins presents a link between lower MM and a child’s insecure attachment [26–28]. Several interventions have been developed to enhance maternal MM, including video feedback and smartphone apps, all of which have demonstrated promise across diverse caregiver populations [29–31]. Importantly, it appears that caregivers’ socioeconomic status and child characteristics do not significantly impact MM levels, indicating that these interventions can be broadly applicable regardless of background or individual child traits [26,32]. A relationship has been shown between maternal and paternal MM with child attachment security and theory of mind abilities [24,25].

Based on attachment theory, early experiences with caregivers shape internal working models that affect future relationships [11]. Thus, children who are securely attached are better able to understand different perspectives within relationships [33]. Insecure attachment is a risk factor for internalising problems such as anxiety [34,35] while secure attachment is associated with appropriate mind-related comments and fewer non-attuned comments [27,36]. Previous studies have indicated that parent cognitions may play a vital role in mediating the relationship between parent and child difficulties [22,37–39]. Some research suggests that MM might be a better predictor than the sensitivity of attachment status [40], at least be a prerequisite for sensitivity [41]. Therefore, focusing on MM in interventions may improve outcomes for families.

Maternal MM has been found to be predictive of children’s ToM, the ability to understand and attribute mental states to oneself and others [42–44]. Mothers who describe their children in more mental terms have children who perform better on tasks that require an understanding of false beliefs [28,45]. Moreover, higher ToM abilities are linked to secure attachment in preschoolers [46,47]. On the other hand, poor ToM abilities are associated with childhood anxiety disorders and negative interpretive biases [48,49].

This study will focus on examining whether training caregivers in MM can reduce social anxiety in preschoolers by improving attachment security and ToM abilities. Understanding these pathways from MM to childhood anxiety can help inform preventative interventions targeting early socio-cognitive development. Therefore, the proposed mechanisms in this study are attachment and the theory of mind. MM strongly predicts child attachment security [41], which provides an anxiety buffer. Insecure attachment is linked to increased internalising symptoms [35]. Caregiver’s MM also forecasts children’s theory of mind abilities [50].

Parental mentalization (MM) is strongly linked to secure attachment in children. Interventions that target MM could improve attachment security. Future research should prioritise MM as a key intervention target to address gaps in how attachment behaviours are passed from parent to child, especially when parents struggle to understand and respond to their child’s emotional needs. Consistent parental behaviours, such as emotional responsiveness, promote secure attachment in children. Interventions, such as training programmes to enhance parents’ ability to perceive and interpret their child’s emotions, can help bridge these gaps, supporting children’s emotional and social development [24,36,51,52].

Various interventions have been developed to enhance MM, ranging from video feedback to smartphone apps, demonstrating promise in diverse caregiver populations [29–31]. Upon closer examination of these interventions, it becomes evident that their effectiveness largely depends on addressing the unique needs of caregivers. Factors such as accessibility, ease of use, and the relevance of the content play crucial roles in determining the success of each intervention format. By enhancing caregivers’ MM, they are better equipped to cultivate secure attachments and establish a nurturing environment, and they can significantly decrease the symptoms of social anxiety in their children.

Various interventions have been conducted by researchers to explore the link between child development and caregivers’ mind-related comments. Schacht et al.(2017) reported that a single-session video-feedback intervention was effective in reducing non-attuned comments [31]. This intervention proved to be beneficial for mother-infant interactions even up to the second year of life. Another study conducted by Larkin et al. developed the BabyMind app, which was successful in increasing MM among adolescent mothers [30]. Walker’s (2012) research emphasised the importance of MM in preschool clinical interventions [53].

This paper proposes an RCT of MM training for caregivers of socially anxious preschoolers. This study emphasises the need to adapt interventions for diverse populations. Research indicates culture shapes caregiving approaches, parenting behaviours, and attachment styles [54,55]. Some studies reveal differences in MM across Western and Eastern cultures. For instance, British parents exhibit higher MM towards children compared to Chinese parents, but both show positive links between MM and child theory of mind [56]. Research indicates that Western mothers engage in more mental state talk with children than Eastern mothers [57]. Collectivist values in Eastern cultures prioritise hierarchy and conformity versus individuality in the West impacts manifestations of MM in parent-child interactions [58,59]. According to some research, traditional gender norms can also affect father-daughter relationships and emotional attunement in Persian families specifically [60,61]. For instance, in a study comparing parenting practices in the UK and India, they found notable differences: UK mothers made more mind-minded comments, while Indian mothers issued more instructions and positive comments, reflecting cultural priorities [62]. These differences highlight the influence of cultural contexts on parenting practices. Recognising and understanding these variations can guide the development of interventions sensitive to cultural contexts, ultimately promoting healthier outcomes for children worldwide [54,63].

## 2. Materials and methods

### 2.1. Objectives

The main aim of this study is to evaluate the efficacy of the caregiver’s MM training in reducing social anxiety symptoms among preschool children. Additionally, to examine how culture (Iranian and British) moderates the relationship between caregivers’ MM training and reductions in social anxiety symptoms among preschool children.

### 2.2. Trial design

**Fig 1:**
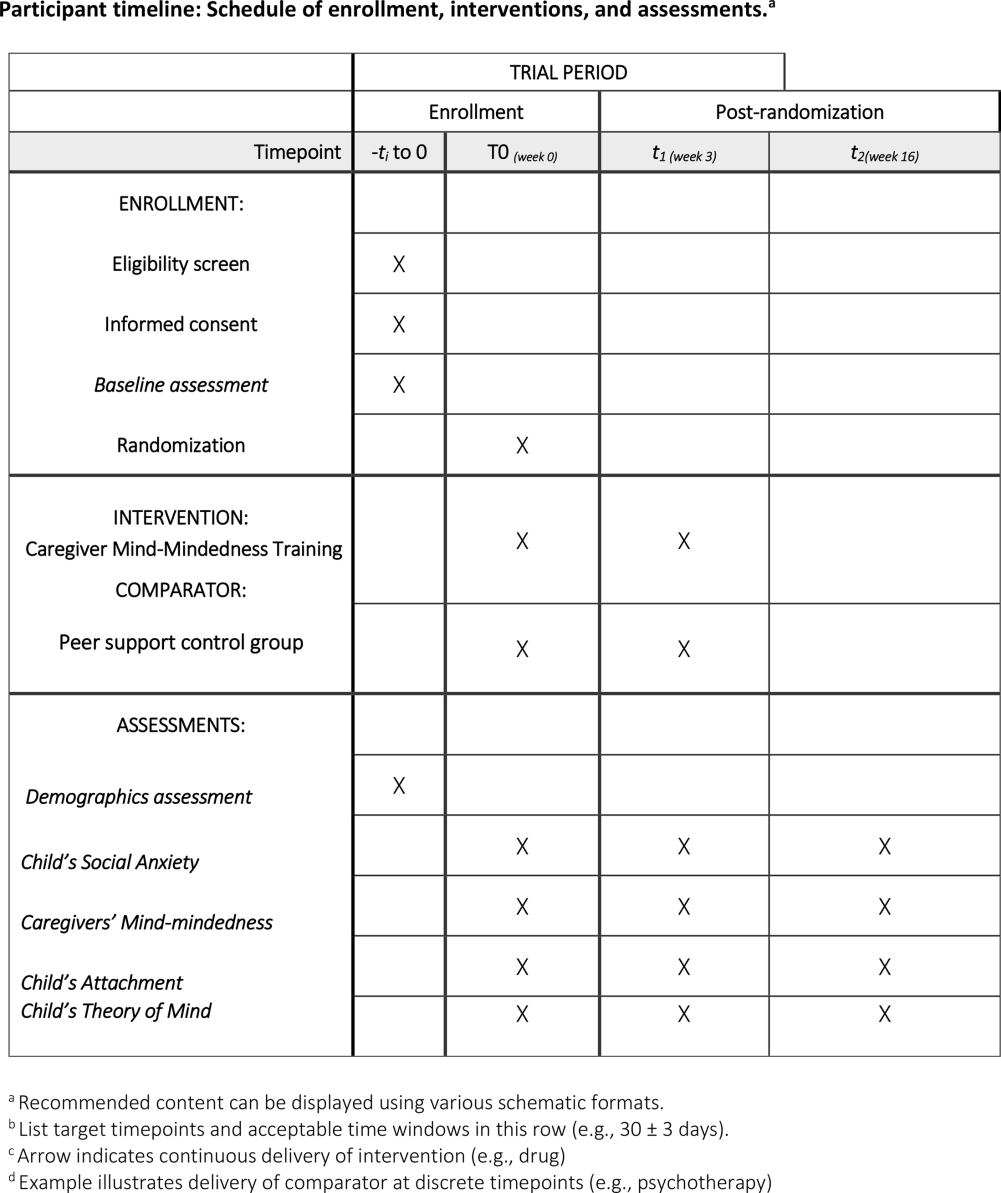
SPIRIT.

This study protocol outlines the design and methodology of a randomised controlled trial aimed at investigating whether MM training for caregivers can effectively reduce social anxiety symptoms in preschoolers by employing a cross-cultural approach involving caregivers from the UK and Iran. Participants will be randomly assigned to either the MM training intervention group or a peer support (control) group. Randomisation will be carried out separately for each country’s sample. Stratified random sampling will ensure a balanced allocation within each country’s participants. The schedule of enrollment, interventions, and assessments is presented in Fig 1.

### 2.3. Study setting

The study will be conducted in two countries: the United Kingdom and Iran. The intervention and all measurements will be held online.

### 2.4. Participants

The study aims to recruit 100 primary caregivers (aged 18–60 years) of preschool-aged children (aged 4–7 years) who exhibit symptoms of social anxiety. Eligible participants must reside in either the United Kingdom or Iran. Caregivers whose children have existing clinical diagnoses or are currently receiving other forms of treatment or support will not be included in the study. Children will be included in the study based on symptoms of social anxiety reported by caregivers, assessed using the Spence Children’s Anxiety Scale – Parent Version (SCAS-P). Although the SCAS was initially validated for children aged 6–12 [64], it has been reliably utilised with younger children aged 4–6 in parent-report studies (Edwards et al., 2010). For this study, the inclusion criteria will specifically focus on elevated scores in the social phobia subscale. Those whose anxiety profiles indicate separation anxiety or general behavioural inhibition, rather than social anxiety, will be excluded during the screening process. Children with a confirmed clinical diagnosis, such as autism spectrum disorder, generalised anxiety disorder, or ADHD, along with those currently receiving psychological or educational treatment, will not be eligible for participation. The research team will not make any formal diagnoses; inclusion decisions will be based solely on parent-reported data obtained through the SCAS-P. This approach guarantees consistency across participants while focusing on observable symptoms rather than clinical labels.

Participants will be recruited through a combination of targeted outreach and community engagement strategies. This includes advertisements in preschools and nurseries, referrals from early years professionals, and word-of-mouth recommendations from other caregivers. The recruitment strategy is designed to ensure a demographically diverse yet diagnostically homogeneous sample of families, with equal representation from the UK and Iran.

Due to the nature of the intervention, participant blinding is not possible, as caregivers will be fully informed of their group allocation, either participating in mind-mindedness training sessions or joining a peer-support group. However, the assessors responsible for data collection and analysis, including those evaluating mind-mindedness, child social anxiety, attachment, and theory of mind, will remain blinded to the group allocations to ensure an unbiased evaluation of outcomes.

To enhance participant retention and reduce attrition, several strategies will be implemented. These will include clear communication protocols, flexible scheduling options, and modest incentives for completing assessments. Participants will receive systematic reminders about their sessions, and a dedicated support structure will be available throughout the duration of the study. Additionally, data from participants who deviate from the protocol will be included in the intention-to-treat analysis. This approach will help preserve the integrity of the randomisation process while minimising bias and ensuring that the findings are practically applicable and generalizable.

**Table:** 

Inclusion Criteria	Primary caregiver of a child aged 4–7 years Caregiver aged 18–60 years Living in the UK or Iran Observable social anxiety symptoms in children confirmed by a screening tool
Exclusion Criteria	Child with another clinical diagnosis Child receiving other support/treatment

### 2.5. Procedure

**Fig 2:**
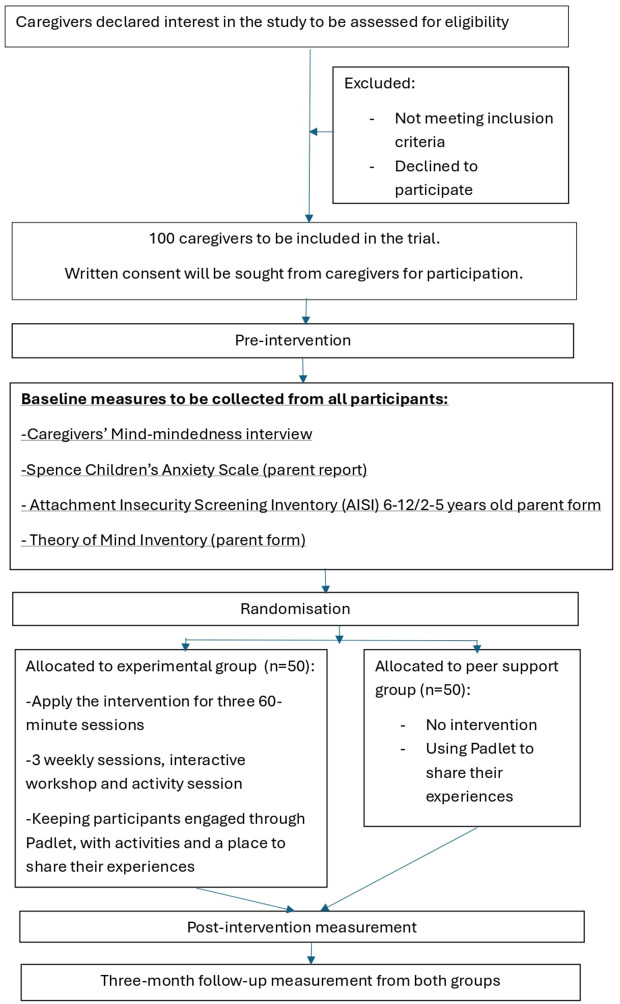
RCT design.

Participant allocation will be carried out using a computer-generated randomisation sequence through Random Allocation Software 2.0, which will assign caregivers to either the mind-mindedness (MM) training intervention group (n = 50) or the peer support control group (n = 50). The randomisation will be stratified by country (UK and Iran) to maintain a balanced allocation within each cultural context (Fig 2).

**Fig 3:**
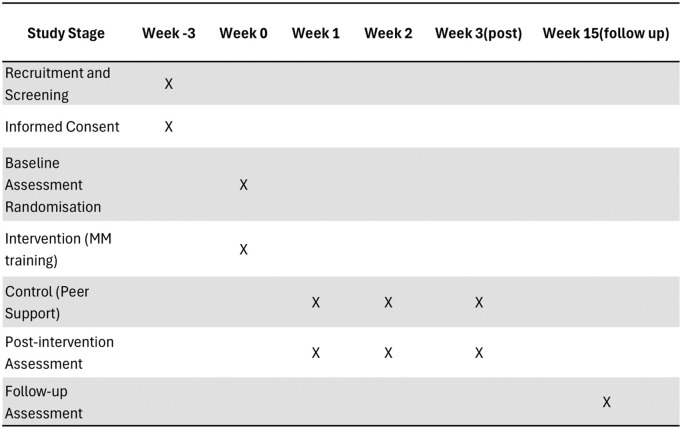
“Participants’ Timeline”.

Data collection will take place at three time points: baseline (T0), post-intervention (T1), and 3-month follow-up (T2). At each time point, data will be collected through validated caregiver-report measures (Fig 3). Mind-mindedness will be assessed using the Mind-Mindedness Coding Manual 2.2 [65], which offers a structured framework for coding caregivers’ descriptions of their children. This method evaluates both the frequency and appropriateness of the mental-state language caregivers use when describing their child. It has been widely applied in developmental studies involving preschool-aged children. Child social anxiety symptoms will be measured using the Spence Children’s Anxiety Scale – Parent Version (SCAS-P) [66], while the scale was initially validated for children aged 6–12, it has also been effectively used in parent-report studies with younger children aged 4–6 [67]. Attachment security will be assessed using the Attachment Insecurity Screening Inventory (AISI) for Preschoolers [68]. This caregiver-report tool is specifically designed for two age groups: children aged 2–5 years and 6–12 years old, which evaluates both anxious and avoidant attachment patterns. The AISI has demonstrated good construct validity and cross-cultural applicability, making it suitable for use in both the UK and Iranian populations.

The assessment of Theory of Mind (ToM) will be conducted using the Theory of Mind Task Battery [69] for evaluating the theory of mind. The battery has been validated for use with preschool children and demonstrates strong convergent validity with other socio-cognitive measures. All measures were selected based on their age appropriateness, prior validation within preschool populations, and their cross-cultural applicability.

Additionally, demographic information (such as age, gender, socio-economic status, and cultural background) will be collected to describe the study population and control for potential confounding variables. Programme adherence will be monitored through attendance records and weekly caregiver reflections on their experiences.

The informed consent process will follow institutional guidelines, ensuring participants receive detailed study information in their preferred language (English or Persian). Participants will have opportunities to ask questions and will receive a clear explanation of their right to withdraw from the study. The Principal Investigator (PI) is responsible for obtaining informed consent from all potential trial participants.

To enhance engagement in the online format, participants will receive regular reminders via email and SMS before each session. The sessions will be interactive and will include breakout room role-plays and guided reflection activities. The control group will encourage informal peer support and provide a platform for them to share their experiences.

Comprehensive data protection measures will be implemented, including secure storage of consent forms and assessment data in encrypted, password-protected files. Unique participant identifiers will be assigned, and access will be restricted to authorised research personnel only. All data management procedures will comply with GDPR and local data protection regulations. The final dataset will be accessible only to the research team and will be stored according to Heriot-Watt University’s data retention policy. This thorough approach to randomisation, data collection, and protection ensures both scientific validity and participant confidentiality throughout the study.

### 2.6. Intervention

This study aims to reduce social anxiety in preschoolers by tailoring an MM intervention that targets parents to alleviate social anxiety levels in their children. Collaborating with parents and caregivers will result in a more effective and tailored solution that resonates with their practical needs and experiences. Their input can lead to a more feasible and impactful study design, refining eligibility criteria, recruitment, and data collection procedures. A participatory co-design was conducted in order to strengthen the quality and outcomes of this research. The intervention will be identical in both countries, with the only difference being the language used for the programme. All other elements, such as the structure, content, delivery methods, and materials of the MM training programme, will remain uniform across both locations.

The MM training programme will consist of three online sessions, each lasting one hour and conducted over three consecutive weeks. The training sessions will be conducted through Zoom, and participants will receive a unique link to access the platform securely. This training is designed to equip caregivers with strategies for attuned, mentalizing caregiving, focusing on enhancing their ability to understand and respond to their child’s emotional and mental states.

The first session will introduce the concept of mind-mindedness and its importance in caregiving. It will include psychoeducation on the development of social anxiety in children and how caregiver responsiveness plays a crucial role. Caregivers will also be introduced to basic emotion coaching techniques, which will be explored in more depth in the second session. The second session will focus on teaching and practising these emotion coaching techniques, helping caregivers recognise, validate, and guide their children’s emotions in a supportive manner. The final session will deepen the caregivers’ understanding through reflective discussions and advanced role-play scenarios that address common challenges in caregiving with socially anxious children.

All three sessions will include video examples demonstrating effective and ineffective approaches, as well as role-play exercises where caregivers can practice these techniques with guided feedback. The role-plays will be led by trained child psychologists. They will give immediate feedback and answer questions to ensure that caregivers are effectively practising mentalizing caregiving. Throughout the training, caregivers will receive support materials and reminders to track their progress and reflections. Participation will be tracked through attendance records and the completion of reflection activities to make sure that participants are following the programme guidelines.

### 2.7. Control group

The peer support (control) group will access a private online group for the exchange of experiences and coping strategies related to the child’s anxiety, without direct MM training. Control group participants will access a secure online forum, where they can post entries and interact through comments, called Padlet. No Zoom sessions will be used, and no structured training or psychoeducation will be delivered in this group.

The comprehensive set of measures includes assessments of MM, child anxiety scales, and evaluations of attachment and theory of mind. Measures of MM will be coded from caregiver descriptions of the child using the MM Coding Manual 2.2 [65]. Child anxiety will be assessed with the Spence Children’s Anxiety Scale [66]. Attachment will be measured using the Attachment Insecurity Screening Inventory [68]. Theory of mind will utilise the Theory of Mind Task Battery [70]. The three-day MM training programme incorporates psychoeducation, role-play exercises, and practical sessions, while the peer support (control) group serves as an active control condition.

### 2.8. Statistical methods

#### 2.8.1. Sample size and power.

A power analysis was conducted in advance using G*Power 3.1 to determine the necessary sample size for linear regression models with two predictors: group assignment and baseline score. Assuming a medium effect size (f² = 0.15), an alpha level of 0.05, and a power of 0.80, the analysis indicated that a minimum sample size of 89 participants is required. To account for an expected attrition rate of approximately 10–15%, we plan to recruit 100 participants, with 50 participants in each group. These participants will be evenly distributed between the United Kingdom and Iran. This target is deemed feasible and suitable for an early-phase efficacy trial. The sample is designed to provide sufficient power for testing the primary effects of the intervention using regression-based methods, while maintaining enough power for within-group analyses.

#### 2.8.2. Primary analysis.

The primary analysis will evaluate the effectiveness of the mind-mindedness training in improving outcomes for both caregivers and children immediately after the intervention. We will use linear regression models for each outcome, which includes caregiver mind-mindedness, child social anxiety (using SCAS-P), attachment insecurity (AISI), caregivers’ MM, and the Theory of Mind (ToM Task Battery). Each model will compare the post-intervention scores (Time 1) between the intervention and control groups, while controlling for baseline values (Time 0). All analyses will adhere to intention-to-treat principles. The effects of the intervention will be assessed using linear regression models, comparing outcomes at post-intervention (T1) and follow-up (T2) to baseline (T0), while controlling for baseline scores. When applicable, multiple imputation with 20 datasets will be used to address any missing data. Effect sizes will be reported using standardised beta coefficients and 95% confidence intervals.

#### 2.8.3. Secondary analyses.

Secondary analyses will assess the persistence of intervention effects at the three-month follow-up (Time 2). Linear regression models will compare follow-up scores to baseline scores while controlling for baseline values. This analysis will provide evidence regarding the durability of the intervention outcomes.

Mediation Analysis: Path analysis using structural equation modelling will test whether attachment security and theory of mind mediate the relationship between MM training and child anxiety reduction. Model fit will be assessed using standard indices (CFI > 0.95, RMSEA < 0.06, SRMR < 0.08).

Moderation analyses will investigate the influence of cultural context (UK vs. Iran) by including interaction terms in multiple regression models. Additionally, exploratory correlational analyses will examine the relationships between changes in mind-mindedness, social anxiety, attachment, and Theory of Mind. In these analyses, effect sizes and confidence intervals will be reported alongside p-values. To manage the risk of Type I and Type II errors, false discovery rate (FDR) corrections will be applied using the Benjamini-Hochberg procedure where appropriate.

#### 2.8.4. Data management.

Data will be collected electronically using REDCap, ensuring standardised collection and secure storage. Quality assurance measures will include double data entry verification for 20% of cases, automated range checks, weekly backups, and monthly data integrity audits to identify patterns of missing data. All analyses will be conducted using Stata 17.0 (StataCorp, College Station, TX) with a significance level of p < 0.05 (two-tailed). Results will be reported following CONSORT guidelines for randomised trials.

#### 2.8.5. Safety monitoring.

Given that the intervention is non-clinical, educational in nature, and conducted remotely, the study is deemed low risk. Consequently, a formal data safety monitoring board is not required. Nevertheless, the research team will maintain oversight of participant welfare throughout the duration of the trial. Any adverse events or indications of distress will be documented and reported in accordance with established ethical standards. Participants will also be reminded of their right to withdraw from the study at any time without facing any repercussions.

### 2.9. Trial status

Ethical approval for the study was granted by the Social Sciences Ethics Committee of Heriot-Watt University in September 2024. Recruitment will commence on 16th September 2024. This study is expected to be completed by February 2025. Prospectively registered on ClinicalTrials.gov (ID: NCT06657014; registered on 23rd October 2024) and on the Iranian Registry of Clinical Trials (IRCT) Version 1 (ID: 80088; approved on 2nd November 2024).

## 3. Discussion

This study will contribute to the understanding of MM in parenting and its potential as a clinical intervention for preschoolers with social anxiety. The literature underscores the need for experimental trials to determine whether interventions in MM can effectively reduce social anxiety, given the current lack of such studies, while also acknowledging the associations between mentalization, attachment, and theory of mind.

The rationale behind this study lies in the existing literature that highlights the crucial role of MM in fostering positive socioemotional outcomes in children.This study builds upon this foundation, intending to provide valuable insights into the effectiveness of an MM training programme for caregivers of preschoolers with social anxiety. The envisioned randomised controlled trial design incorporates a comprehensive curriculum, including psychoeducation, role-play exercises, and practical sessions, with the aim of enhancing caregivers’ ability to recognise and appropriately respond to their child’s internal states. While the outcomes of the trial remain to be seen, the study’s design aligns with the intent of offering an innovative early intervention approach.

The cross-cultural component of the study is particularly noteworthy. The cross-cultural investigation enriches the study by exploring societal and cultural influences on MM, with implications for the development of culturally sensitive family-based treatments. Comparing an individualistic Western culture (UK) to a more collectivist Middle Eastern culture (Iran) will provide new insights into cultural variations in MM and impacts on intervention effectiveness. Exploring culture as a moderator can inform culturally sensitive applications [45,71–75]. The co-design process, involving caregivers in shaping the intervention, adds a participatory dimension that can enhance the relevance and impact of the study.

While the content of the intervention remains consistent across both the UK and Iran, cultural factors may impact its reception and effectiveness. For instance, in Iran, collectivist parenting norms may prioritise obedience and respect for authority, potentially influencing how mind-minded practices are viewed in terms of appropriateness and intuitiveness [76–78]. Conversely, UK caregivers may find it easier to engage with the intervention’s emphasis on individualised mental state talk, given the nation’s values surrounding autonomy and emotional expression [45].

Potential cultural resistance or reinterpretation of MM practices may affect both engagement and efficacy, underscoring the importance of cultural sensitivity in the delivery of training. Although the sessions are linguistically tailored, future research could explore more profound cultural adaptations based on these contextual factors.

The research has potential implications for clinical practice and future research. If the trial demonstrates positive outcomes, it could pave the way for integrating MM training into existing parenting programmes as a targeted approach to alleviate social anxiety symptoms in preschoolers. Additionally, the study’s findings may prompt further exploration of the cultural variations in parental reflections on children’s internal states and their implications for intervention design. The findings are expected to inform clinical practice and contribute to the growing body of research on early interventions for childhood mental health. As previous studies (see [32] for a review) suggest links between MM and enhanced attachment security and theory of mind, the training may alleviate social anxiety by strengthening these protective socio-emotional factors. However, direct evidence establishing MM training as an anxiety intervention is limited. The proposed MM training programme, if proven effective, could offer a valuable addition to the arsenal of strategies aimed at promoting positive mental health outcomes in preschoolers. The study’s design, cultural sensitivity, and participatory approach in shaping the intervention position it as a potential catalyst for advancing research and clinical practices in the realm of child mental health.

## Supporting information

S1 Appendix Study materials. (DOCX)

S1 Protocol Full study protocol. (DOCX)

S1 Checklist SPIRIT checklist. (DOC)
