# Supplementary material for: Caregiver mind-mindedness training as an early intervention for social anxiety in children: A protocol for a randomised controlled trial
Source: PLoS One. 2025 Sep 22;20(9):e0315150. doi: 10.1371/journal.pone.0315150 (PMC12453178; doi:10.1371/journal.pone.0315150)
Supplement: S1 Appendix — (DOCX) [file pone.0315150.s001.docx]

# Appendix 1

## 1.Information Sheet

PARTICIPANT INFORMATION SHEET

PROJECT TITLE: Caregiver Mind-Mindedness training as an Early Intervention for Socially Anxious Children

INVITATION

You are being invited to take part in a research study for an intervention for children's social anxiety. You must be the primary caregiver, responsible for the daily care of a child aged 4-7 with social anxiety symptoms, age 18-50. You must also have access to the internet. The research is supervised by Daniel Hale, Mary E. Stewart and Minu Mathews. The investigator is Hiva Javadian. The project has been approved by the School of Social Sciences Ethics Committee at Heriot-Watt University.

WHAT WILL HAPPEN

If you decide to participate, you will first have a brief interview lasting up to 30 minutes, which will be audio recorded, and 3 questionnaires to fill in. Following this, you will be randomly assigned to one of two groups:

1- Mind-Mindedness (MM) Training Group

Participants in this group will attend a training programme designed to enhance their understanding and responsiveness to their child's emotional and mental states. The training includes psychoeducation, emotion coaching, video examples, reflective discussions, and role-play exercises. The aim is to promote attuned and mentalizing caregiving behaviours.

Commitment: Three 1-hour sessions held over three weeks.

2- Peer Support Group

Participants in this group will have access to a private online platform. This space allows caregivers to share experiences and strategies related to managing their child's anxiety, fostering a supportive community environment.

Commitment: Time spent engaging with the platform will vary based on personal participation.

Regardless of your group, you will need to undergo assessments before the intervention, immediately after the intervention, and three months after the intervention. These assessments, which will each take about an hour, are designed to measure mind-mindedness, your child's anxiety levels, attachment, and theory of mind.

Note: If you are assigned to the peer support group, you will still receive the complete set of training materials used in the mind-mindedness training group after the three-month follow-up assessment. This ensures that all participants have access to the valuable content of the training, regardless of their initial group assignment.

AIM OF THE PROJECT

The aim of the study is to investigate the effectiveness of caregivers’ mind-mindedness training in reducing social anxiety symptoms among preschool-aged children. By assessing the impact of a targeted intervention on caregiver behavior and its effects on child social anxiety, this research aims to contribute to the development of early interventions for enhancing child well-being and promoting mental health outcomes. Moreover, this study will explore the potential role of attachment and theory of mind as underlying mechanisms for the observed changes in social anxiety symptoms. This study will focus on how cultural differences between Iran and the UK may moderate this relationship.

What is Mind-Mindedness?

Mind-mindedness refers to the ability to recognize and consider a child's thoughts, feelings, and intentions. In this study, Mind-Mindedness training aims to improve caregivers' ability to understand and respond to their child's inner experiences, which may help reduce the child's social anxiety.

Please be aware that participants will be assigned randomly to either the Mind-Mindedness training or the peer support group to ensure a fair and unbiased evaluation of the interventions.

TIME COMMITMENT

Mind-Mindedness Training Group: Attend three 1-hour training sessions over three weeks.

Assessments: Complete assessments at three time points (baseline, post-intervention, three-month follow-up), each taking about one hour.

Peer Support Group: Engage with the online platform, with the time commitment varying based on your level of participation.

Your participation in this study is greatly appreciated. It could make a significant difference in understanding and addressing social anxiety in young children.

PARTICIPANTS’ RIGHTS

You may decide to stop being a part of the research study at any time without explanation. You have the right to ask that any data you have supplied to that point be withdrawn until the data is fully anonymised. Full anonymisation of your data will only happen once the data has been analysed. You have the right to omit or refuse to answer or respond to any question that is asked of you.

BENEFITS AND RISKS

There are no known benefits or risks for you in this study.

COST, REIMBURSEMENT AND COMPENSATION

Your participation in this study is voluntary; there is no compensation for your participation in this study

PRIVACY AND CONFIDENTIALITY

All data will be fully anonymized during the transcription process. Any identifying details, such as names, addresses, and places of work, will be removed or destroyed to ensure confidentiality.

Heriot-Watt University is the data controller for the personal data collected in this project. We will collect and use your personal data for this project only with your consent. You can withdraw your consent at any time until the data is fully anonymised by contacting the researcher, supervisor or the data protection team.

We will keep your personal data securely and ensure that no one will link the research data you provide to any identifying information you may supply. Once we have analysed the information you provide, we will completely anonymise your personal data so that it will not be possible to identify you from any information in the remaining dataset. After the project ends, we may use the anonymous dataset for research outputs such as articles and conference presentations.

If you would like to know more about what Heriot-Watt University does with your personal data and your rights under privacy law, please visit our data protection web pages at https://www.hw.ac.uk/uk/services/information-governance/protect/privacy-and-your-data-rights.htm or contact our Data Protection Officer by email at dataprotection@hw.ac.uk.

FOR FURTHER INFORMATION

Daniel Hale will be glad to answer your questions about this study and provide additional information on results if requested. You may contact them at [d.hale@hw.ac.uk](mailto:d.hale@hw.ac.uk).

## 2. Participant Consent Form

By signing below, you are agreeing that:

• You have read and understand the Participant Information Sheet and Consent Form

• You understand that there are no expected potential risks to you in your participation

• You are taking part in this research study voluntarily (without coercion or remuneration).

• You consent for any personal data1 collected to be used as part of this study

• Any questions you may have about your participation in this study have been answered satisfactorily

Personal data refers to information in any format about an individual who can be identified directly or indirectly from that information. It includes but is not restricted to factual information such as date of birth, ID number, or location and may include sensitive information such as health, ethnicity and opinions expressed. Once personal data has been completely anonymised so that it is impossible to re-identify the individuals concerned it ceases to be personal data. The University may use anonymised data as summary statistics or for analyses but only where it is truly anonymous.

DATE ______________________________________________

[I Agree]

[I Do Not Agree]

## 3. Debrief Sheet

PROJECT TITLE: CAREGIVER MIND-MINDEDNESS TRAINING AS AN EARLY INTERVENTION FOR SOCIALLY ANXIOUS CHILDREN

INVESTIGATORS

We are a group of researchers studying children’s social anxiety. The research is supervised by Daniel Hale, Mary E. Stewart and Minu Mathews. The investigator is Hiva Javadian.

INTRODUCTION

Childhood social anxiety, with onset in preschool years (Hitchcock et al., 2009; Mohammadi et al., 2020), has been linked to attachment style and deficits in theory of mind (ToM) (Cristina Colonnesi et al., 2017; Ronchi et al., 2020). Caregiver-child interactions and parental mentalisation like mind-mindedness influence socio-emotional development and attachment security (Meins, 1997; Meins et al., 2003), with higher mind-mindedness promoting positive outcomes (Bernier et al., 2023; Fishburn et al., 2022; Miller et al., 2019). Culture shapes parenting and attachment styles (Kamza, 2019; Strand et al., 2019; Zaidman-Mograbi et al., 2020). COVID-19 increased child anxiety (Moore et al., 2020; Racine et al., 2021), underscoring the need for youth mental health resources. Insecure attachment and ToM deficits can contribute to social anxiety (Bowlby, 1969), while low caregiver attunement through poor mind-mindedness may exacerbate it (Meins, Centifanti, et al., 2013). Examining links between low caregiver mind-mindedness and childhood social anxiety (Meins, 2001) can inform early intervention to alleviate symptoms and prevent disorders, critical for child well-being (Courtney et al., 2020; Ng & Ng, 2022).

AIM OF STUDY

The aim of the study was to investigate the effectiveness of caregivers’ mind-mindedness training in reducing social anxiety symptoms among preschool-aged children. By assessing the impact of a targeted intervention on caregiver behavior and its effects on child social anxiety, this research aimed to contribute to the development of early interventions for enhancing child well-being and promoting mental health outcomes. Moreover, this study explored the potential role of attachment and theory of mind as underlying mechanisms for the observed changes in social anxiety symptoms. This study focused on how cultural differences between Iran and the UK might have moderated this relationship.

PROCEDURE

The study aimed to recruit 100 primary caregivers of socially anxious preschool-aged children, aged 4-7 years, from the UK and Iran. Recruitment methods included distributing flyers at preschools/nurseries, word-of-mouth, and referrals from teachers. Participants had a short max 30-minutes interview and then were randomly assigned to either the mind-mindedness training group (n=50) or the peer support group (n=50), using a computer-generated random sequence and allocation concealment to ensure unbiased assignment.

The mind-mindedness training group underwent a 3-day (1h) online training programme, which included psychoeducation, emotion coaching, video examples, reflective discussions, and role-play exercises aimed at enhancing attuned, mentalizing caregiving behaviours. The peer support group had access to a private online platform for sharing experiences and coping strategies related to their child's anxiety, without direct mind-mindedness training.

Data collection occurred at baseline, post-intervention, and 3-month follow-up using validated measures.

FOR FURTHER INFORMATION

Daniel Hale will be glad to answer your questions about this study and provide additional information on results if requested. You may contact them at d.hale@hw.ac.uk.

PRIVACY AND CONFIDENTIALITY

All data will be fully anonymized during the transcription process. Any identifying details, such as names, addresses, and places of work, will be removed or destroyed to ensure confidentiality.

Heriot-Watt University is the data controller for the personal data collected in this project. We are using your personal data for this project only with your consent. You can withdraw your consent at any time until the data is fully anonymised by contacting the researcher, supervisor or the data protection team.

We will keep your personal data securely and ensure that no one will link the research data you provide to any identifying information you may supply. Once we have analysed the information you provide, we will completely anonymise your personal data so that it will not be possible to identify you from any information in the remaining dataset. After the project ends, we may use the anonymous dataset for research outputs such as articles and conference presentations.

If you would like to know more about what Heriot-Watt University does with your personal data and your rights under privacy law, please visit our data protection web pages at https://www.hw.ac.uk/uk/services/information-governance/protect/privacy-and-your-data-rights.htm or contact our Data Protection Officer by email at dataprotection@hw.ac.uk.

REFERENCES

Bowlby, J. (1969). Attachment and loss: Volume I: Attachment. Basic Books.

Courtney, D., Watson, P., Battaglia, M., Mulsant, B. H., & Szatmari, P. (2020). COVID-19 impacts on child and youth anxiety and depression: Challenges and opportunities. The Canadian Journal of Psychiatry, 65(10), 688-691. https://doi.org/10.1177/0706743720935646

Fishburn, S., Meins, E., Fernyhough, C., Centifanti, L. C. M., & Larkin, F. (2022). Explaining the relation between early mind-mindedness and children’s mentalizing abilities: The development of an observational preschool assessment. Developmental Psychology, 58(1), 17-31. https://doi.org/10.1037/dev0001272

Hitchcock, C. A., Chavira, D. A., & Stein, M. B. (2009). Recent findings in social phobia among children and adolescents. Israel Journal of Psychiatry and Related Sciences, 46(1), 34-44.

Kamza, A. (2019). Attachment to mothers and fathers during middle childhood: An evidence from Polish sample. BMC Psychology, 7(1), Article 79. https://doi.org/10.1186/s40359-019-0361-5

Meins, E. (1998). The effects of security of attachment and material attribution of meaning on children's linguistic acquisitional style. Infant Behavior and Development, 21(2), 237-252. https://doi.org/10.1016/S0163-6383(98)90004-2

Meins, E., Centifanti, L. C., Fernyhough, C., & Fishburn, S. (2013). Maternal mind-mindedness and children's behavioral difficulties: Mitigating the impact of low socioeconomic status. Journal of Abnormal Child Psychology, 41(4), 543-553. https://doi.org/10.1007/s10802-012-9699-3

Meins, E., Fernyhough, C., Fradley, E., & Tuckey, M. (2001). Rethinking maternal sensitivity: Mothers’ comments on infants’ mental processes predict security of attachment at 12 months. Journal of Child Psychology and Psychiatry, 42(5), 637-648. https://doi.org/10.1111/1469-7610.00759

Mohammadi, M. R., Salehi, M., Khaleghi, A., Hooshyari, Z., Mostafavi, S. A., Ahmadi, N., Hojjat, S. K., Safavi, P., & Amanat, M. (2020). Social anxiety disorder among children and adolescents: A nationwide survey of prevalence, socio-demographic characteristics, risk factors and co-morbidities. Journal of Affective Disorders, 263, 450-457. https://doi.org/10.1016/j.jad.2019.11.167

Ng, C. S. M., & Ng, S. S. L. (2022). Impact of the COVID-19 pandemic on children's mental health: A systematic review. Frontiers in Psychiatry, 13, Article 975936. https://doi.org/10.3389/fpsyt.2022.975936

Racine, N., McArthur, B. A., Cooke, J. E., Eirich, R., Zhu, J., & Madigan, S. (2021). Global prevalence of depressive and anxiety symptoms in children and adolescents during COVID-19: A meta-analysis. JAMA Pediatrics, 175(11), 1142-1150. https://doi.org/10.1001/jamapediatrics.2021.2482

Ronchi, L., Banerjee, R., & Lecce, S. (2020). Theory of mind and peer relationships: The role of social anxiety. Social Development, 29(2), 478-493. https://doi.org/10.1111/sode.12417

Strand, P. S., Vossen, J. J., & Savage, E. (2019). Culture and child attachment patterns: A behavioral systems synthesis. Perspectives on Behavior Science, 42(4), 835-850. https://doi.org/10.1007/s40614-019-00220-3

Zaidman-Mograbi, R., le Roux, L., & Hall, H. (2020). The influence of culture on maternal attachment behaviours: A South African case study. Children Australia, 45(1), 30-39. <https://doi.org/10.1017/cha.2020.4>

## 4. Audio Recording consent form

FOR AUDIO RECORDING

Heriot-Watt University is the data controller for the personal data that you have consented to be collected in this project. We will keep your personal data securely and ensure that no one will link the research data you provide to any identifying information you may supply.

We are using your personal data for this project only with your consent. You can withdraw your consent at any time until the data is fully anonymised contacting the researcher, supervisor or the data protection team.

Once this recording has been transcribed or analysed, the recording will be erased and your data will be anonymised.

As a participant in this study, I agree to be audio-recorded for the purpose of collecting data for this study as well as a means of verifying results from other data collected. I am aware that I may withdraw this consent at any time without penalty, at which point, the audio recording will be erased. I understand that once this recording has been transcribed or analysed, the recording will be erased and my data will be anonymised.

PARTICIPANT

DATE ______________________________________________

[I Agree]

[I Do Not Agree]

5. Flyer

6. Data Collection Instruments

This section contains standardised instruction for caregivers’ mind-mindedness(MM), which cannot be reproduced here due to copyright restrictions. Interested researchers may contact the test publishers for access.

This section contains a standardised instrument for attachment security (AISI), which cannot be reproduced here due to copyright restrictions. Interested researchers may contact the test publishers for access.

This section contains a standardised instrument for children’s theory of mind (ToM Task Battery), which cannot be reproduced here due to copyright restrictions. Interested researchers may contact the test publishers for access.

This section contains the standardised instrument for children's social anxiety, the Spence scale (SCAS), which cannot be reproduced here due to copyright restrictions. Interested researchers may contact the test publishers for access.

7. Ethics Approval Letter
